# Gene expression patterns in hypoxic and post-hypoxic adult rat retina with special reference to the NMDA receptor and its interactome

**Published:** 2009-02-09

**Authors:** Lori Ann Crosson, Roger A. Kroes, Joseph R. Moskal, Robert A. Linsenmeier

**Affiliations:** 1 Department of Biomedical Engineering, Northwestern University, Evanston, IL; 2 The Falk Center for Molecular Therapeutics, Northwestern University, Evanston, IL; 3 Department of Neurobiology & Physiology, Northwestern University, Evanston, IL

## Abstract

**Purpose:**

A gene expression analysis of hypoxic rat retina was undertaken to gain a deeper understanding of the possible molecular mechanisms that underlie hypoxia-induced retinal pathologies and identify possible therapeutic targets.

**Methods:**

Rats were made severely hypoxic (6%–7% O_2_) for 3 h. Some rats were sacrificed at this time, and others were allowed to recover for 24 h under normoxic conditions. A focused oligonucleotide microarray of 1,178 genes, qRT–PCR of selected transcripts, and western analysis of hypoxia inducible factor-1α (HIF-1α) were used to compare retinas from the hypoxic and recovery groups to control animals that were not made hypoxic. SAM analysis was used to identify statistically significant changes in microarray data, and the bioinformatics programs GoMiner, Gene Set Enrichment Analysis (GSEA), and HiMAP were used to identify significant ontological categories and analyze the N-methyl-D-aspartate (NMDA) receptor interactome.

**Results:**

HIF-1α protein, but not mRNA, was elevated up to 15-fold during hypoxia, beginning at 0.5 h, the shortest duration examined. Of the total of 1,178 genes examined by microarray, 119 were significantly upregulated following hypoxia. Of these, 86 were still significantly upregulated following recovery. However, 24 genes were significantly downregulated following hypoxia, with 12 still significantly downregulated after recovery. Of the 1035 genes that did not change with hypoxia, the expression of 36 genes was significantly changed after recovery. Ontological analyses showed significant upregulation of a large number of genes in the glutamate receptor family, including 3 of the 5 NMDA subunits. qRT–PCR analysis further corroborated these findings. Genes known to directly interact specifically with the NR1 subunit of the NMDA receptor were identified using HiMAP databases. GSEA analysis revealed that these genes were not affected by either hypoxia or altered after recovery.

**Conclusions:**

The identification of gene expression alterations as a function of hypoxia and recovery from hypoxia is important to understand the molecular mechanisms underlying retinal dysfunction associated with a variety of diseases. Gene changes were identified in hypoxic retina that could be linked to specific networks. Retinas recovering from hypoxia also showed distinct patterns of gene expression that were different from both normoxic control retinas and hypoxic retinas, indicating that hypoxia initiates a complex pattern of gene expression. Diseases of which hypoxia is a component may exhibit the several changes found here. Several potential therapeutic targets have been identified by our approach, including modulation of NMDA receptor expression and signaling, which until now have only been shown to play a role in responding to ischemia.

## Introduction

Evidence continues to accumulate that retinal tissue hypoxia is an important intermediate step in the pathogenesis of many retinal diseases. In most cases, hypoxia is caused by either a dysfunction of the retinal vasculature, as in diabetic retinopathy and retinal artery and vein occlusions, or it is due to a mismatch between nutrient supply and demand, as in the case of a retinal detachment, where the retina is separated too far from the choroid to receive sufficient oxygen. Some evidence of the involvement of retinal tissue hypoxia has come from in vivo measurements in animal models of disease, though the onset of hypoxia and the severity of the insult have been difficult to identify. Linsenmeier et al. [1] measured intraretinal PO_2_ with microelectrodes in cats with long-standing diabetes and concluded that the retina was hypoxic in the early stages of retinopathy. Harris et al. [2] also provided evidence of the presence of retinal hypoxia in the early stages of diabetes by showing that contrast sensitivity was improved when patients with early diabetic retinopathy were made hyperoxic. Further evidence of hypoxia is suggested by the increases in vascular endothelial growth factor (VEGF) expression in patients with diabetic retinopathy [3–5] and retinopathy of prematurity (ROP) [6], because VEGF is known to be a hypoxia-inducible gene [7,8]. Hypoxia is also implicated in glaucoma by the finding of elevated hypoxia-inducible factor [9]. Experimental hypoxia rapidly reduces photoreceptor oxidative metabolism and increases glycolysis [10–13]. Chronic experimental hypoxia kills photoreceptors in adult rat retina [14] and can lead to retinal angiogenesis [15].

Little is known about the molecular effects of hypoxia on the retina and the underlying relationship between hypoxia and the pathogenesis of retinal diseases. Conventional studies investigating molecular effects of hypoxia in the retina have focused on one particular pathway or one particular class of molecules at a time. For example, hypoxia has been reported to induce VEGF expression in the retina, both at the mRNA [16,17] and protein levels [16]. Another important molecule, the transcription factor hypoxia inducible factor-1 (HIF-1), is well established as a key molecular signal linking oxygen level to gene expression in many tissues. HIF-1 activation has been observed in oxygen-induced ischemic retinopathy [17] and in hypoxia [16]. In addition, temporal [17] and spatial [16,17] correlations between increased levels of HIF-1α, the regulatory subunit of HIF-1, and increased VEGF expression have been shown, indicating that HIF-1 may control the expression of VEGF in the retina under hypoxic conditions.

The advent of microarray technology, with its capacity to monitor the expression of thousands of genes simultaneously, has provided a novel opportunity to identify individual genes, groups of genes, and related “gene families” associated with a given biologic process. Microarray-based gene profiling has been used to examine many aspects of retina biology and pathology. Gene expression patterns in normal adult or the developing retina have been reported for mouse [18–23], aged and normal adult human [24–26], canine [27], and rabbit [28]. Farkas et al. [29] and Ivanov et al. [30] reported on gene expression profiling in purified populations of rat retinal ganglion cells. Trimarchi et al. [31] performed single cell expression profiling of developing murine ganglion and amacrine cells.

To date, only one microarray-based study examining the effects of hypoxia on retinal gene expression has been reported [32]. In the present study, we therefore used microarray technology to investigate the concurrent expression of 1,178 genes in the rat retina both following hypoxia and following a post-hypoxic 24 h reoxygenation period. GoMINER [33,34], Gene Set Enrichment Analysis (GSEA) [35] and Human Interactome Mapping (HiMAP) were then applied to identify those gene families that showed differential expression during hypoxia or recovery.

Of particular interest were the N-methyl-D-aspartate receptor (NMDAR) genes, which were further studied by qRT–PCR. NMDARs are part of the large family of glutamate receptors, and are unique in that they require both glutamate and glycine or serine [36] to become fully active. They play a pivotal role in regulating synaptic transmission, modulating excitotoxicity responsible for much of the neuronal damage caused by hypoxic insult in the brain [37], and are expressed in retinal photoreceptors, horizontal cells, and bipolar cells as well as the amacrine and ganglion cells of the inner retina [38–41]. Hama et al. [42] reported that modulators of the glycine site of NMDAR are markedly enhanced in in vivo, excitotoxin-induced, retinal ganglion cell damage. We found that the expression of distinct NMDAR subunit mRNAs was enriched in both hypoxic and post-hypoxia retina.

## Methods

### Induction of hypoxia and Isolation of tissues

Adult Long-Evans pigmented rats were used in all experiments. Rats were obtained from Harlan Sprague-Dawley (Madison, Wisconsin), maintained on a 12–12 light-dark cycle and had access to standard rat chow and water ad lib. This research included measures to reduce suffering and minimize the total number of animals. It was approved by the Northwestern University IACUC and conformed to the NIH Guide for the Care and Use of Laboratory Animals. Animals were brought to the investigators’ laboratory for the hypoxia exposure and sacrifice. Hypoxia was induced by placing rats in a large Plexiglas chamber in which oxygen content was maintained at 6%–7% using a ProOx oxygen control module (Biospherix, Redfield, NY). During the hypoxic episode, animals were left in their home cages in the hypoxia chamber to minimize stress. For microarray and qRT–PCR measurements (i.e., all molecular measurements except those for HIF-1α message and protein), animals were sacrificed by decapitation without anesthesia either immediately following 3 h of hypoxia or following a 24 h post-hypoxia recovery period in normal air. The rationale for choosing these time points was based on our finding that HIF-1α was maximally upregulated by 3 h, and on previous work in brain [43]. Control animals for all studies were brought to the laboratory and left in their home cages for the same duration as those subjected to hypoxia and were then decapitated. Animals that were allowed to recover from hypoxia were returned to the animal care facility and brought back to the laboratory for sacrifice. Animals used for measurement of HIF-1α mRNA and protein were made hypoxic for variable durations, from 0.5 to 6 h, before sacrifice. A group of 3 h hypoxic animals was allowed to recover for 24 h before sacrifice and measurement of HIF-1α.

Following decapitation, the retinas were rapidly removed by using the method described by Winkler [12]. Briefly, the eyeball was proptosed by placing forceps around the optic nerve close to its exit from the eye. A slit was then made in the cornea using a sharp blade, and the globe was opened. The cornea and lens were then removed. The forceps was then moved toward the eye to increase pressure and gently separate the retina. The retina was then dissected out and immediately frozen at −80 °C. This procedure from decapitation to freezing was completed in less than two minutes.

### Blood gas measurements

Blood gas measurements were made on a group of adult Long-Evans pigmented rats separate from those used in the molecular studies. Anesthesia was induced and maintained using isoflurane. Induction of anesthesia took place in a plastic box placed in the fume hood through which a gas mixture of approximately 5% isoflurane, 35% oxygen and 60% nitrogen was passed. After induction, anesthesia was maintained by use of a nose cone with a rubber diaphragm that fitted tightly around the rat snout and was vented to a snorkel. A tracheal cannula was inserted, and the isoflurane was then administered through the cannula. The percentage of isoflurane was reduced to 2% to maintain surgical anesthesia. A cannula was inserted into the femoral artery for blood pressure recording and measurements of PO_2_, pH, and PCO_2_. During surgery, anesthesia was monitored via toe pinch, muscle relaxation, and respiration. Temperature was maintained at 37–38 °C by a thermostatically controlled water blanket. Once surgery was completed, the rats were exposed to a gas mixture of approximately 2% isoflurane and air, and blood gas measurements were made with a Ciba-Corning 860 Blood Gas Analyzer (Siemens, Norwood, MA). Rats were then exposed to a mixture of approximately 2% isoflurane, 6 to7% oxygen, and 92% nitrogen. Blood gas measurements were similarly recorded.

### mRNA sample preparation and qRT–PCR

Total RNA was extracted from the retinal samples using RNeasy Lipid Tissue Mini Kit (Qiagen, Inc., Valencia, CA). cDNA synthesized by reverse transcription of 1 µg RNA primed with oligo(dT) and random 9-mer primers was used as the substrate for quantitation of mRNA expression levels by quantitative RT–PCR in the presence of SYBR^®^ Green (Stratagene, La Jolla, CA). Gene-specific primers were designed to generate approximately 100 bp amplicons with PerlPrimer software [44] and Primer3 software, or determined from previous reports [45]. Primers were chosen from exons separated by large introns, when possible, and PCR reaction quality and specificity were verified by gel electrophoresis and melting-curve dissociation analysis of the amplified product. Amplification parameters including primer concentrations, and annealing temperatures were optimized for each primer pair. The primers used for quantitative RT–PCR are listed in Table 1. Relative quantification of original input RNA amounts were calculated by comparison to standard curves using purified PCR product as a template for the mRNAs of interest and were normalized to the amount of acidic ribosomal phosphoprotein P0 mRNA [24,46].

**Table 1:** qRT–PCR primers used in the study

**GenBank gene ID**	**Accession number**	**Primer sequence (5′-3′)**	**Primer concentration (μM)**	**Annealing** **temperature (°C)**
Acidic ribosomal phosphoprotein (P0)	NM_022402	F: AGTACCTGCTCAGAACAC	200	55
		R: TCGCTCAGGATTTCAATGG		
Erythropoietin (EPO)	NM_017001	F: CTCAGAAGGAATTGATGTCG	400	55
		R: GGAAGTTGGAGTAGACCC		
Erythropoietin receptor (EPOR)	NM_017002	F: CTCGTCCTCATCTCACTG	400	61
		R: ACCCTCAAACTCATTCTCTG		
N-methyl-D-aspartate receptor 1 (NR1)	NM_017010	F: ATGGCTTCTGCATAGACC	400	59
		R: GTTGTTTACCCGCTCCTG		
N-methyl D-aspartate receptor 2A (NR2A)	NM_012573	F: AGTTCACCTATGACCTCTACC	400	59
		R: GTTGATAGACCACTTCACCT		
N-methyl-D-aspartate receptor 2B (NR2B)	NM_012574	F: AAGTTCACCTATGACCTTTACC	400	59
		R: CATGACCACCTCACCGAT		
N-methyl D-aspartate receptor 2C (NR2C)	U08259	F: GGCCCAGCTTTTGACCTTAGT	400	59
		R: CCTGTGACCACCGCAAGAG		
N-methyl D-aspartate receptor 2D (NR2D)	NM_022797	F: GTTATGGCATCGCCCTAC	600	59
		R: CATCTCAATCTCATCGTCCC		
Vascular endothelial growth factor (VEGF)	NM_031836	F: AGGAAAGGGAAAGGGTCA	400	57
		R: ACAAATGCTTTCTCCGCT		
VEGF receptor 1 (FLT-1)	NM_019306	F: ATAAGAACCCTGATTACGTGAG	400	57
		R: TCACTCTTGGTGCTGTAGAC		
VEGF receptor 2 (FLK-1)	U93306	F: AAGCAAATGCTCAGCAGGAT	400	57
		R: TAGGCAGGGAGAGTCCAGAA		

### Microarray experiments

#### Oligonucleotide probe selection and synthesis

The 1,178 genes comprising the Falk Center for Molecular Therapeutics (FCMT) Rat CNS microarray were compiled from currently available NCBI/EMBL/TIGR rat sequence databases and commercially available central nervous system (CNS) microarrays (Affymetrix, Santa Clara, CA) and provided representation from greater than 90% of the major gene ontological categories [47]. Individual 45-mer oligonucleotides corresponding to mRNAs of each gene were used as probes, and immobilized on microarray slides, as described below. Array Designer software v2.03 (Premier Biosoft International, Palo Alto, CA) was used to select and optimize these oligonucleotides, as described [47]. This optimization was based on combining very stringent selection criteria (minimal secondary structure, minimal homology to other genes in the available rat genomic databases, no low complexity or repeat regions, and defined Tm) with a statistical ranking algorithm [48]. By employing standard phosphoramidite chemistry, we synthesized oligonucleotides with a PolyPlex^TM^ 96 well oligonucleotide synthesizer (GeneMachines^®^, Palo Alto, CA). The oligonucleotide probes were quantitated using a spectrophotometer and immobilized on the microarrays via inclusion of a 5′-amino linker (Glen Research, Sterling, VA) onto each oligonucleotide.

#### Array fabrication

Each microarray was manufactured using an OmniGrid^TM^ robotic microarrayer (GeneMachines). The oligonucleotides, suspended in 1.5 M betaine in 3X SSC buffer at a concentration of 500 ng/µl, were covalently linked in quadruplicate to aldehyde-coated glass microscope slides at a spacing of 250 microns. A four pin configuration was used in printing the slides, and four large subarrays were printed on each slide. After printing, the arrays were baked ad vacuo at 80 °C for 2 h and stored desiccated at room temperature until use.

#### Target preparation and microarray hybridization

Total RNA was extracted from retinal samples using RNeasy Lipid Tissue Mini Kit (Qiagen) and was the substrate for amplification and labeling using a procedure based on the Eberwine protocol [49]. Specifically, reverse transcription of 5 µg total RNA primed with an oligo(dT) primer bearing a T7 promoter was followed by in vitro transcription, generating multiple antisense copies of each mRNA in the sample (Ambion, Austin, TX). Inclusion of modified amino-allyl dUTP at a ratio of 3:2 with unmodified dUTP in the in-vitro transcription reaction generated antisense RNA (aRNA) capable of chemically coupling to amine reactive dyes. aRNA was also prepared from a universal rat reference sample (Stratagene, La Jolla, CA) following the same procedure. Study samples (control, hypoxic, recovery) were labeled with Cy5 dye, and reference samples were labeled with Cy3 dye. After purification, 2 μg of each of the two prepared aRNA samples under study were combined in a hybridization solution containing 8 μg polyd(A), 10 μg rat Cot-1 DNA, 4 μl Liquid Block Solution (Amersham, Piscataway, NJ), 0.2% SDS, and 1.5X SSC in a final volume of 53 μl. The combined samples were subsequently denatured and hybridized to the microarrays in a humidified hybridization chamber at 46 °C for 16 h. For each time point, retinal RNA samples from each of 5 rats were studied in triplicate (3 microarray slides per rat). Because each oligonucleotide was spotted in quadruplicate on the array, there were a total of 12 expression measurements per gene in each experimental group.

### Data acquisition

Arrays were scanned using two lasers (633 nm and 543 nm) at 5 μ resolution with ScanArray 4000XL (Packard Biochip Technologies, Billerica, MA) using ScanArray Express software v2.0. Data from these scans were collected as two 16-bit .tiff images. Individual spot intensities from these images were quantitated using median pixel intensity with BlueFuse software (BlueGnome, Cambridge, UK). Along with each of the 16-bit .tiff files relating to both the Cy3 and the Cy5 samples, raw data files containing spot intensity values were uploaded to an in-house server running GeneTraffic (Iobion Informatics, La Jolla, CA). GeneTraffic is a bioinformatics server system for microarray data storage and analysis in Minimum Information about a Microarray Experiment (MIAME) compliant format [50]. Prior to normalization, quality confidence measurements (spot diameter, spot area, array footprint, spot circularity, signal to noise ratio, spot uniformity, background uniformity, and replicate uniformity) were determined for each scanned array on a spot-by-spot basis to assess quality. Spots that did not pass these criteria were not included in further analysis. The mean log_2_ ratios for each spot were normalized using the LOWESS curve-fitting equation on a print-tip specific basis to allow for differences among the four printing pins used during array manufacturing. This method is recommended for routine normalization of cDNA arrays as it corrects the *M*-values for both subarray spatial variation and for intensity-based trends [51,52].

### Significance analysis of microarrays using rank scores

To identify statistically significant differentially expressed genes, statistical analyses were performed using the permutation-based significance analysis of microarrays using rank scores (SAM-RS) [53] based within the traditional SAM software package (v2.0, Stanford University, Palo Alto, CA) [53]. In our analyses, appropriately normalized data were analyzed using two-class, unpaired analysis on a minimum of 500 permutations and was performed by comparing expression data derived from experimental versus control retinas. The cutoff for significance in these experiments was set at a false discovery rate (FDR) of approximately 5%.

### GoMiner

Traditionally, microarray results have been analyzed using a gene-by-gene approach. In this study, genes identified as differentially expressed by SAM analysis were examined for their biologic association to the gene ontology (GO) categories [34] as defined by the GO Consortium [33]. This provides both additional statistical stringency to the identified genes and identifies groups of related genes or “gene families” which were modulated following hypoxia. Analyses were performed using the ontological mapping software GoMiner. This software calculated the enrichment or depletion of individual ontological categories with genes that had changed expression and identified cellular pathways potentially relevant to hypoxia. Pathways within three independent functional hierarchies, namely biologic process, molecular function, and cellular component, were queried.

### Gene set enrichment analysis

Gene Set Enrichment Analysis (GSEA) is a microarray data-mining technique used to determine whether there is coordinated differential expression or “enrichment” in a set of functionally related genes when comparing control and experimental samples [35]. In the current study, sets of a priori, user-defined functionally related genes were input into GSEA as well as normalized gene expression data from both control versus hypoxia and control versus recovery microarray analyses. The gene sets used were identical to the categories defined by the GO project and used in the GoMiner Analysis. Initially, genes were ranked in a list by the d-statistic as determined by SAM. To determine whether members of a particular gene set were enriched at the top of the list, a Kolmogorov–Smirnov (K-S) running statistic summation was then computed beginning with the top-ranking gene. This running sum increased when a gene defined as belonging to a particular gene set was encountered; otherwise, the sum decreased. The enrichment score (ES) for a single set was then defined as the greatest positive deviation of the running sum across all genes. The ES was computed for every gene set using actual data and the maximum ES (MES) over all of the gene sets was recorded. To determine the significance of these changes, phenotype labels were randomized, genes were reordered and the ES values were recomputed. Permutations were then performed 1,000 times, and a histogram of the corresponding enrichment scores, ES_NULL_, was generated. An estimate of the nominal p-value was calculated as the fraction of the 1,000 random permutations in which the top pathway gave a larger ES result than that observed in the actual data.

### HIF-1α protein sample preparation and western blot analysis

The same methods of inducing hypoxia were used to study HIF-1α protein and message as for the microarray studies, but variable durations of hypoxia, from 0.5 h to 6 h, were used in different rats. Levels of HIF-1α message were determined by qRT–PCR, and protein levels were determined with western blots. For protein measurements, retinal samples were isolated and immediately frozen at −80 °C. Nuclear protein extracts were prepared as previously described [54]. Briefly, retinal samples were homogenized at 4 °C in buffer containing 10 mM Tris (pH 7.4), 1 mM EDTA, 0.15 M sodium chloride, 0.5% NP-40, with protease inhibitors (20 µg/ml aprotinin, 5 µg/ml leupeptin, and 1 mM phenylmethane sulfonyl fluoride-PMSF). The lysates were then incubated on ice at 4 °C for 30 min and centrifuged for 5 min at 500x g at 4 °C. The nuclear pellet was resuspended in a second lysis buffer containing 50 mM HEPES (pH 7.9), 0.4 M sodium chloride, and 1 mM EDTA, with protease inhibitors (20 µg/ml aprotinin, 5 µg/ml leupeptin, and 1 mM PMSF). The suspension was then centrifuged for 5 min at 20,000x g at 4 °C. Total protein concentrations were determined using the bicinchonic acid (BCA) colorimetric protein assay system (Pierce Endogen, Rockford, IL). Sample absorbances were read at a wavelength of 562 nm with a spectrophotometer and compared to a standard curve. HIF-1α protein expression was measured by electrophoresing 100 μg of nuclear extracts on 12.5% SDS-polyacrylamide gels. For a positive control, nuclear extracts were prepared from a rat C6 glioblastoma cell line incubated in the presence of CoCl_2_ for 2 h. CoCl_2_ is known to increase HIF-1α protein levels [55]. These CoCl_2_-treated C6 nuclear extracts were concurrently electrophoresed with the rat retinal protein samples. Following electrophoresis, proteins were transferred to polyvinylidene difluoride membrane, and the membrane was blocked with 5.0% nonfat dry milk in 0.1% Tween-20 in Tris buffered saline (TBS) for 1 h. The membrane was then incubated with a 1:500 dilution of mouse monoclonal anti-HIF-1α antibody (Novus Biologicals, Littleton, CO) in 5.0% nonfat dry milk in 0.1% Tween-20 in TBS at 4 °C overnight. Then the membrane was washed 3 times for 10 min in 0.1% Tween-20 in TBS. Next, it was incubated with a 1:2,000 dilution of horseradish peroxidase-conjugated antimouse IgG (Amersham) for 1 h at room temperature in 0.1% Tween-20 in TBS. The membrane was then washed as already described and developed with the ECL Chemiluminescence detection system (Amersham) according to the manufacturer’s instructions. Protein expression levels were quantitated using the software Scion Image (Scion Corporation, Frederick, MD).

### Statistics

Statistically significant differences among control, hypoxic and recovery values were determined for (a) select gene expression values, as measured by qRT–PCR and (b) among time points for HIF-1α mRNA and protein expression. Comparisons were made by ANOVA followed by the Tukey procedure for posthoc comparisons.

## Results

### Generation of in vivo retinal hypoxia

Arterial P_a_O_2_ was measured in a subset of rats before and immediately following exposure to 6%–7% O_2_, as described in the Methods. The severe systemic hypoxemia reduced arterial P_a_O_2_ from 84.3±11.0 mmHg during air breathing to 20.3±1.2 mmHg during hypoxia (n=3 rats). Retinal PO_2_ has not been measured during hypoxia in rats, but this arterial PO_2_ has been shown to reduce retinal PO_2_ in cats [10].

### HIF-1α protein and mRNA

**Figure 1:**
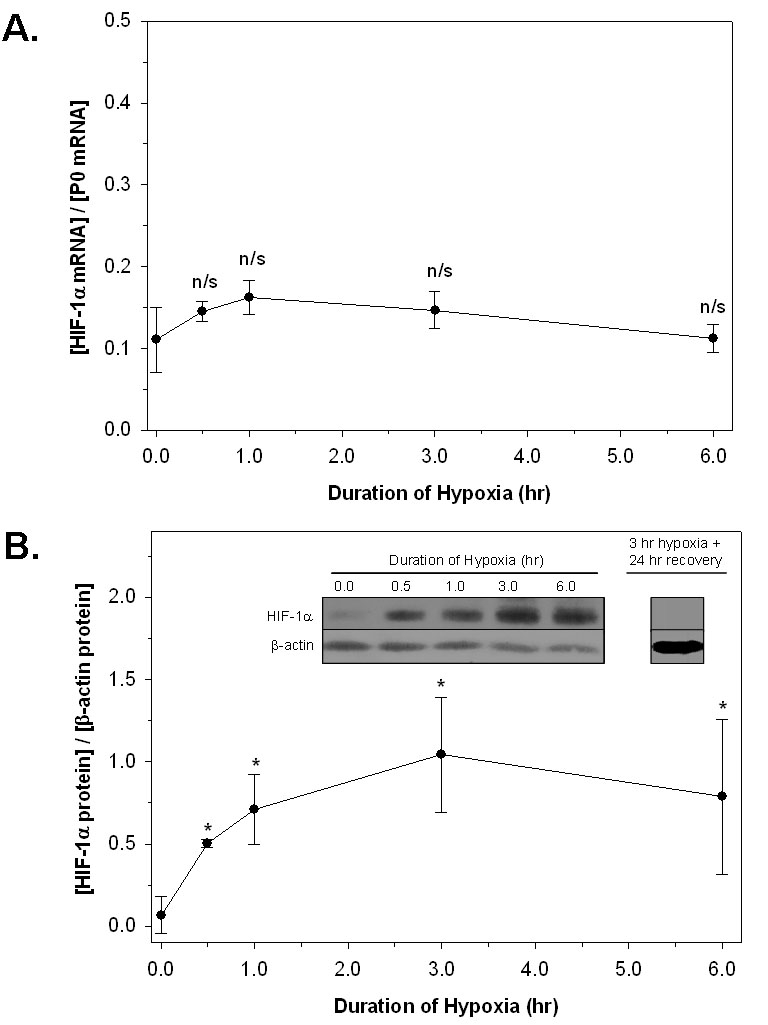
HIF-1α mRNA and protein expression in rat retina during hypoxia. Rats were exposed to 6%–7% O_2_ (hypoxia) for varying durations up to 6 h. **A:** HIF-1α mRNA abundance, normalized to acidic ribosomal protein P0 mRNA, was calculated by qRT–PCR. Data represent mean (±SD) of 3 independent experiments. No significant differences (n/s; p>0.05) were observed between control and hypoxic HIF-1α mRNA expression levels at any time point. **B:** Quantitation of HIF-1α protein expression in nuclear protein extracts by western analysis. Data represent mean (±SD) of at least 3 independent experiments and were normalized to β-actin expression levels. HIF-1α protein levels were higher in all hypoxic samples relative to controls (ANOVA followed by post-hoc test; p<0.05) but were not different between any two hypoxic time points. The inset depicts the western result from a representative time course experiment, as well as the return of HIF-1α protein levels to control levels 24 h after exposure to 3 h hypoxia.

Figure 1B shows that after 0.5 h of hypoxia, HIF-1α protein levels (normalized to levels of β-actin protein expression) were elevated approximately sevenfold relative to the control. HIF-1α protein continued to increase to approximately 15-fold more than the control level after 3 h of hypoxia. A statistically significant increase was observed relative to control at all hypoxic time points up to the 6 h duration of hypoxia. The average HIF-1α protein level was somewhat smaller after 6 h of hypoxia than after three hours, but this decrease was not significant. HIF-1α returned to control levels after 24 h of recovery from 3 h of hypoxia (not shown). This is consistent with results of Bernaudin et al. [43], who reported no difference in HIF-1α protein levels in the neonatal rat brain after a 24 h reoxygenation period compared to control.

HIF-1 α mRNA levels were measured by quantitative RT–PCR analyses at the same time points (Figure 1A). Data were normalized to acidic ribosomal phosphoprotein P0 mRNA levels [24,46]. Northern blotting analyses were performed on control and 3 h hypoxic samples to confirm that mRNA levels for P0 remained unchanged during hypoxia. No significant differences were observed between HIF-1α mRNA levels in control animals and hypoxic animals at any time point. Thus, as in other tissues, it is likely that control of HIF-1α in the retina is largely posttranscriptional, probably at the level of protein turnover.

### Expression of known hypoxia-associated mRNAs

**Figure 2:**
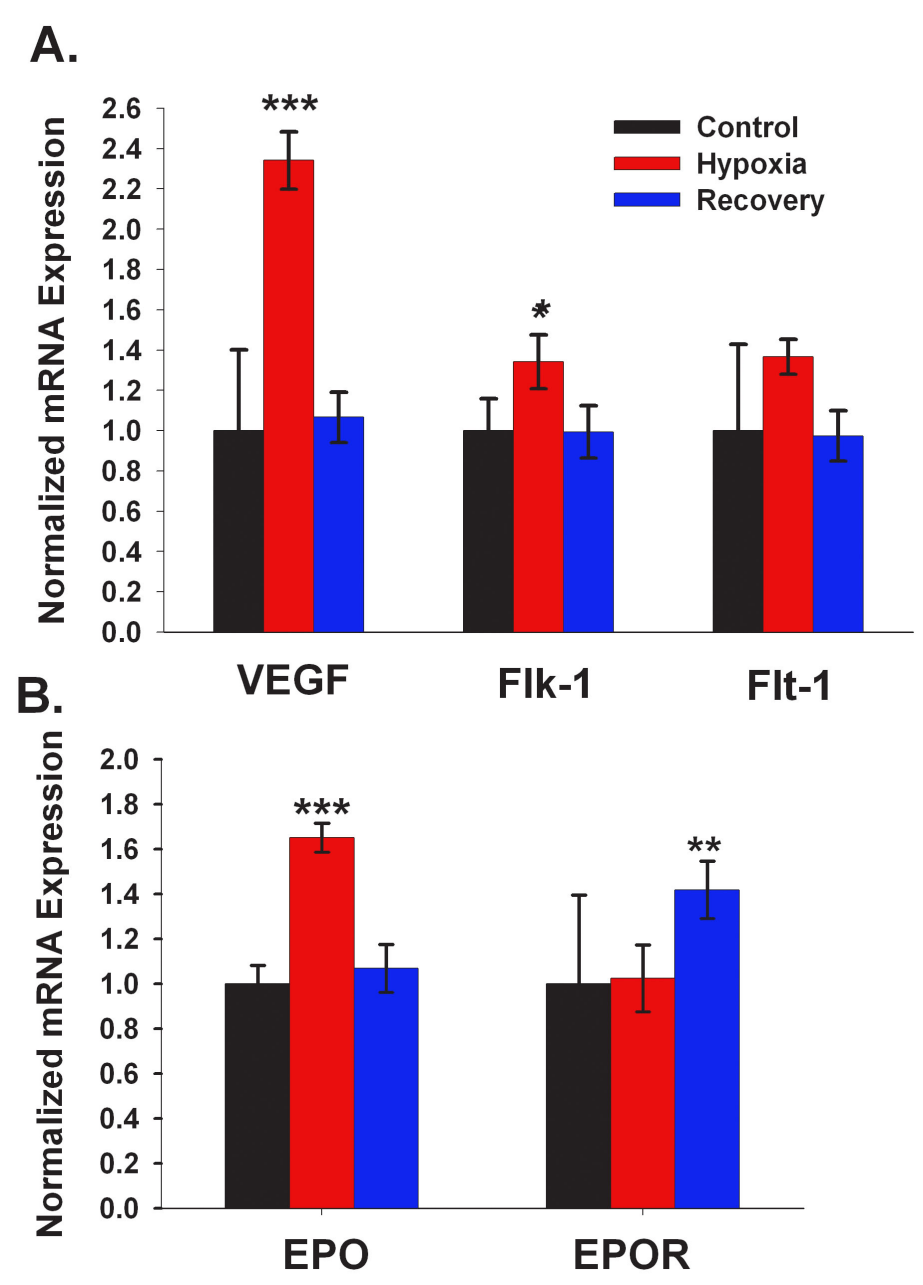
qRT–PCR analysis of known hypoxia-associated mRNAs. For each mRNA, transcript abundance, normalized to acidic ribosomal protein P0, was calculated by qRT–PCR. Values were then further normalized to the control level of each transcript. Data represent mean (±SD) of 5 independent experiments. **A:** The expression of *VEGF* and VEGF receptors *Flk-1* and *Flt-1* mRNA was measured in the rat retina immediately following 3 h of hypoxia and after 24 h of recovery in air. VEGF and Flk-1 were significantly higher in hypoxic samples as compared to control. Flt-1 tended to increase during hypoxia, but the difference from control was not significant. **B:** The expression of *erythropoietin (EPO)* and *erythropoietin receptor (EPOR)* mRNA was measured in the rat retina immediately following 3 h of hypoxia and after 24 h of recovery in air. EPO mRNA levels were significantly higher in hypoxic samples as compared to control. A significant difference was also observed for the EPOR control and hypoxia mRNA levels between hypoxic and recovery samples. The asterisks indicate significance levels assessed via ANOVA followed by post-hoc tests: * - p<0.05; ** - p<0.01; *** - p<0.001.

The expression of a few retinal mRNAs known to be induced by hypoxia, including HIF-1α, was assessed by qRT–PCR analysis of total RNA isolated from retinal tissue following exposure to 3 h of 6%–7% O_2_. Additionally, expression of these mRNAs was also assessed following 24 h of recovery from hypoxia in room air compared to that in retinas isolated from control animals exposed only to air (Figure 2). These included genes associated primarily with the vascular changes resulting from oxygen deprivation; namely (a) *VEGF* and its receptors *Flk-1* and *Flt-1* [17,56]; and (b) *erythropoietin* (*EPO*) and its receptor (*EPOR*) [16]. The expression of *VEGF*, *Flk-1*, and *EPO* were significantly upregulated following hypoxia. The expression of *EPOR*, although unchanged immediately following hypoxia, was significantly upregulated relative to control after 24 h of recovery.

### Gene expression analyses: Identification of hypoxia-associated genes

To detect novel changes in gene expression, we used a focused microarray platform to evaluate the expression profile of retinas from hypoxic animals compared to that in retinas isolated from control animals exposed to room air. Analyses were performed with 5 animals per group (control, 3 h of hypoxia, and 24 h or recovery after 3 h hypoxia) to decrease bias that may be introduced by donor-specific gene expression patterns. As described in the Methods, a reference experimental design was used. RNA samples were studied in triplicate (i.e., 3 microarray slides for each retina).

**Figure 3:**
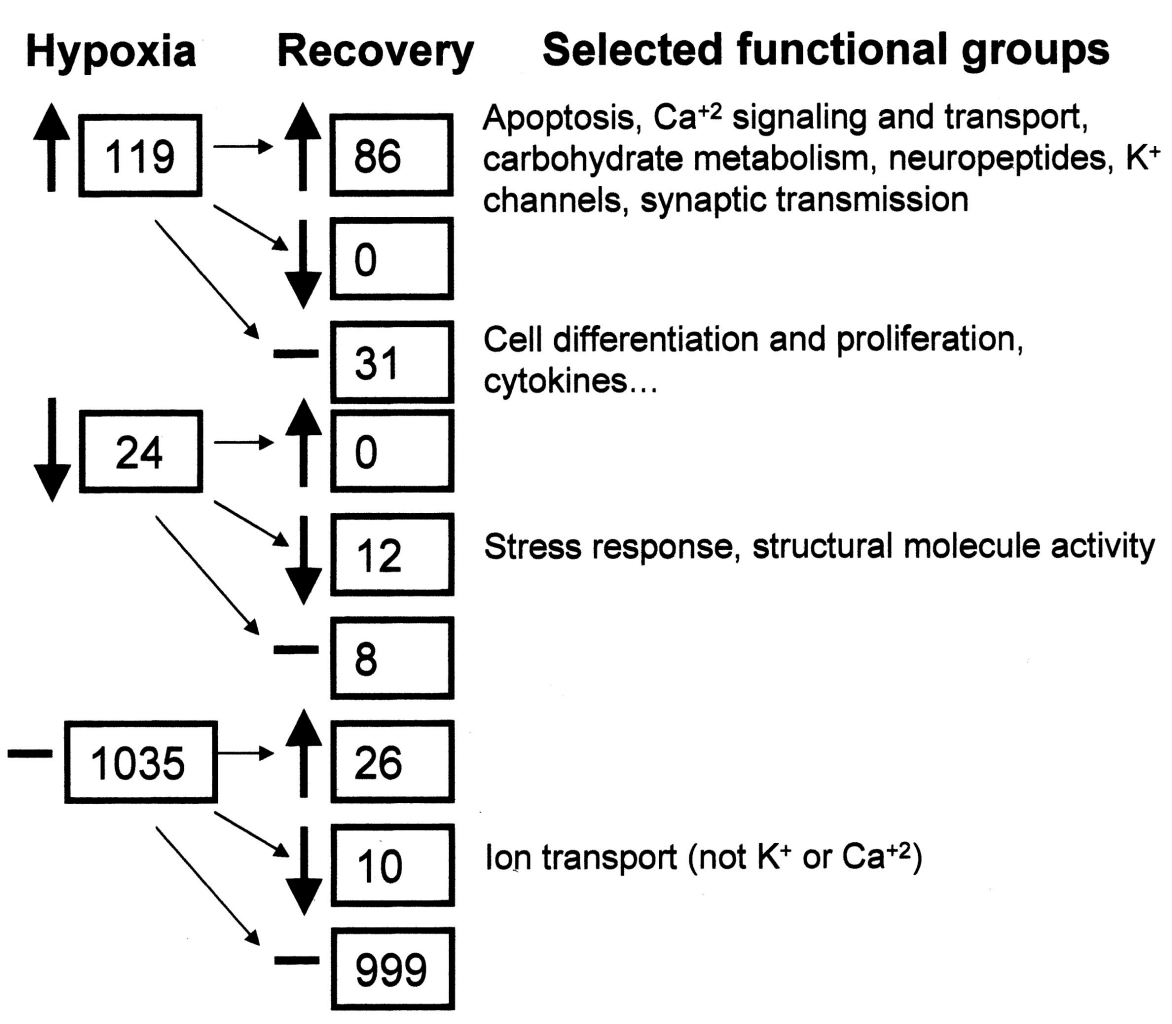
Summary of microarray data. Shown is an examination of the microarray databased on the number of genes upregulated, downregulated, or not changed. These gene changes were categorized into major functional groups. Upward arrows indicate numbers of genes that were significantly upregulated; down arrows indicate those that were downregulated; and horizontal bars represent those that were not altered. The recovery column shows how the genes in each category were affected during recovery. Ellipses indicate that these were not the only categories affected.

Statistical evaluation at a stringent FDR of approximately 5% for the hypoxic-control comparison identified 119 genes whose expression was preferentially upregulated in hypoxic retinas, and 24 that were preferentially downregulated (Figure 3). At this stringent FDR, only 7 of these changes were expected to be false positives. Appendix 1 shows the identities, functional annotations, and relative expression ratios of these genes. Although the abundance ratios of most of these genes were less than twofold, possibly because of the heterogeneity of retinal tissue, significant changes could be detected. Many functional categories of genes were represented in these analyses, including calcium signaling, cytokines, and growth factor pathways. Several of the identified genes were representative of pathways previously described in other tissues exposed to hypoxia, including genes in the stress response, apoptotic, proliferation, and synaptic transmission pathways. Many of the genes known to be directly transactivated by the overexpression of the transcription co-factor HIF-1α were identified, and included *inducible nitric oxide synthetase 2, insulin-like growth factor 1, insulin-like growth factor binding protein 2, and hexokinase 2. VEGF* and its receptors and *erythropoietin* were not on the microarray, but as shown in Figure 2, these were also upregulated. However, many genes and pathways not yet described in the context of hypoxia were also identified.

Changes extending beyond hypoxia itself were also observed when comparing the data sets (Figure 3). Of the 119 genes upregulated following hypoxia, 72% remained upregulated after a 24 h period of recovery under normoxic conditions. The remainder returned to control levels. None of the changes were observed to reverse directionality during the recovery period. Of the 24 genes downregulated following hypoxia, the expression of approximately half remained lower than control levels after recovery. Thus, the majority of the gene expression differences observed persisted for a significant period of time after return to normoxic conditions. As noted above, HIF-1α protein levels returned to normal within this time-frame as did message for *VEGF, Flk-1*, and *EPO* (Figure 2).

### Ontological analyses

#### GOMiner

To provide additional statistical stringency to the identification of potential targets, we then analyzed the data sets generated by the SAM-RS analysis of the microarray data for hypoxia-associated coregulation of multiple, functionally related genes. The genes identified as differentially expressed when comparing hypoxic and control retinal samples and when comparing recovery and control retinal samples at a FDR of approximately 5% were examined for their biologic association to GO categories. Using the GOMiner algorithm, two independent category structures (biologic process and molecular function) based on the 1178 rat genes represented on the microarray (of which 923 currently carry GO annotations) were initially constructed and used as Query gene files. The genes identified in this study were then loaded as a “Query Changed Gene File” into the program to examine the distribution of these genes within the GO category structures. All of these queried genes carried current GO annotations. Table 2 lists the GO categories that were significantly enriched in hypoxia and recovery. Among the most interesting ones identified were those associated with 1) the large category of transport, and the more specific categories of carbohydrate transport and cation:amino acid symport; 2) several related to calcium, including calcium ion binding, calmodulin binding, and voltage gated calcium channel activity; 3) DNA damage response and signal transduction; 4) response to oxidative stress; and 5) oxygen and reactive oxygen species metabolism.

**Table 2:** Gene Ontology categories significantly altered during hypoxia and recovery

**Hypoxia**	**p value**	**Recovery**	**p value**
**Ontological Category**	**Ontological Category**
		**Biologic Process**	
**Biologic Process**		Synaptic Vesicle Endocytosis	0.005
Translation	0.002	Sulfur Metabolism	0.014
Regulation of Translation	0.003	Microtubule Polymerization	0.016
Carboxylic Acid Metabolism	0.014	Cytoskeleton-Dependent Intracellular Transport	0.017
Regulation of Heart Contraction	0.022	Microtubule-Based Movement	0.017
Nitrogen Compound Metabolism	0.023	Oxygen and Reactive Oxygen Species Metabolism	0.018
Carbohydrate Transport	0.026	Regulation of Heart Contraction	0.026
Monosaccharide Metabolism	0.041	DNA Damage Response, Signal Transduction	0.029
Transport	0.041	Microtubule Polymerization or Depolymerization	0.029
		Microtubule-Based Process	0.034
**Cellular Component**		Protein Polymerization	0.034
Membrane-Bound Vesicle	0.003	Microtubule Cytoskeleton Organization and Biogenesis	0.046
Vesicle	0.003	Response to Oxidative Stress	0.049
Golgi Vesicle	0.004		
Cytoplasmic Membrane-Bound Vesicle	0.005	**Cellular Component**	
Chromatin	0.007	Vesicle Membrane	0.002
Nuclear Chromosome	0.007	Intracellular Non-Membrane-Bound Organelle	0.003
Chromosome	0.008	Condensed Chromosome	0.016
Intracellular Non-Membrane-Bound Organelle	0.013	Integral to Membrane of Membrane Fraction	0.016
Condensed Chromosome	0.014	Tubulin	0.016
Integral-to-Membrane of Membrane Fraction	0.014	Voltage-Gated Calcium Channel Complex	0.023
Perinuclear Region	0.026	Cytoskeleton	0.034
Mitochondrial Membrane	0.03	Organelle Membrane	0.035
Organelle Envelope	0.034	Cytosol	0.043
Clathrin-coated Vesicle	0.043	Microtubule	0.046
Envelope	0.043	Protein Complex	0.047
Vesicle Membrane	0.043		
		**Molecular Function**	
**Molecular Function**		Amino Acid Binding	0.034
Amino Acid Binding	0.032	Calmodulin Binding	0.041
Cation:Amino Acid Symporter Activity	0.032	Voltage-Gated Calcium Channel Activity	0.049
Sugar Transporter Activity	0.032	Calcium Ion Binding	0.05
Structural Molecule Activity	0.048		

The genes identified as differentially expressed when comparing hypoxic and control retinal samples and when comparing recovery and control retinal samples were examined for their biologic association to GO categories (detailed in the text). Using GOMiner software, three independent category structures (biologic process, cellular component, and molecular function) were initially constructed and the genes identified in this study were examined for their distribution within these three GO category structures. The significance of the calculated enrichment in each GO category was calculated as a p-value using Fisher's Exact Test. A p<0.05 was considered significant.

#### Gene set enrichment analysis

A limitation of GO analysis is that the predetermined categories may not match the ones of functional interest in a particular tissue. The list in Appendix 1 suggested that some categories that we defined, particularly related to synaptic function, were not GO categories, so GSEA analysis, which allows the creation of additional categories, was employed. GSEA demonstrated significant enrichment in NMDAR subunit expression both during hypoxia and following recovery. χ^2^ analysis was used to determine both the extent and significance of *NMDAR* enrichment as compared to what would be expected by chance alone. As compared to normoxic controls, a statistically significant enrichment of 5.9-fold (χ2=7.750, p=0.0054, 2-tailed) was found in hypoxic retinas and 6.3-fold (χ2=8.432, p=0.0037, 2-tailed) was found in recovery retinas.

### NMDA-R qRT–PCR corroboration

**Figure 4:**
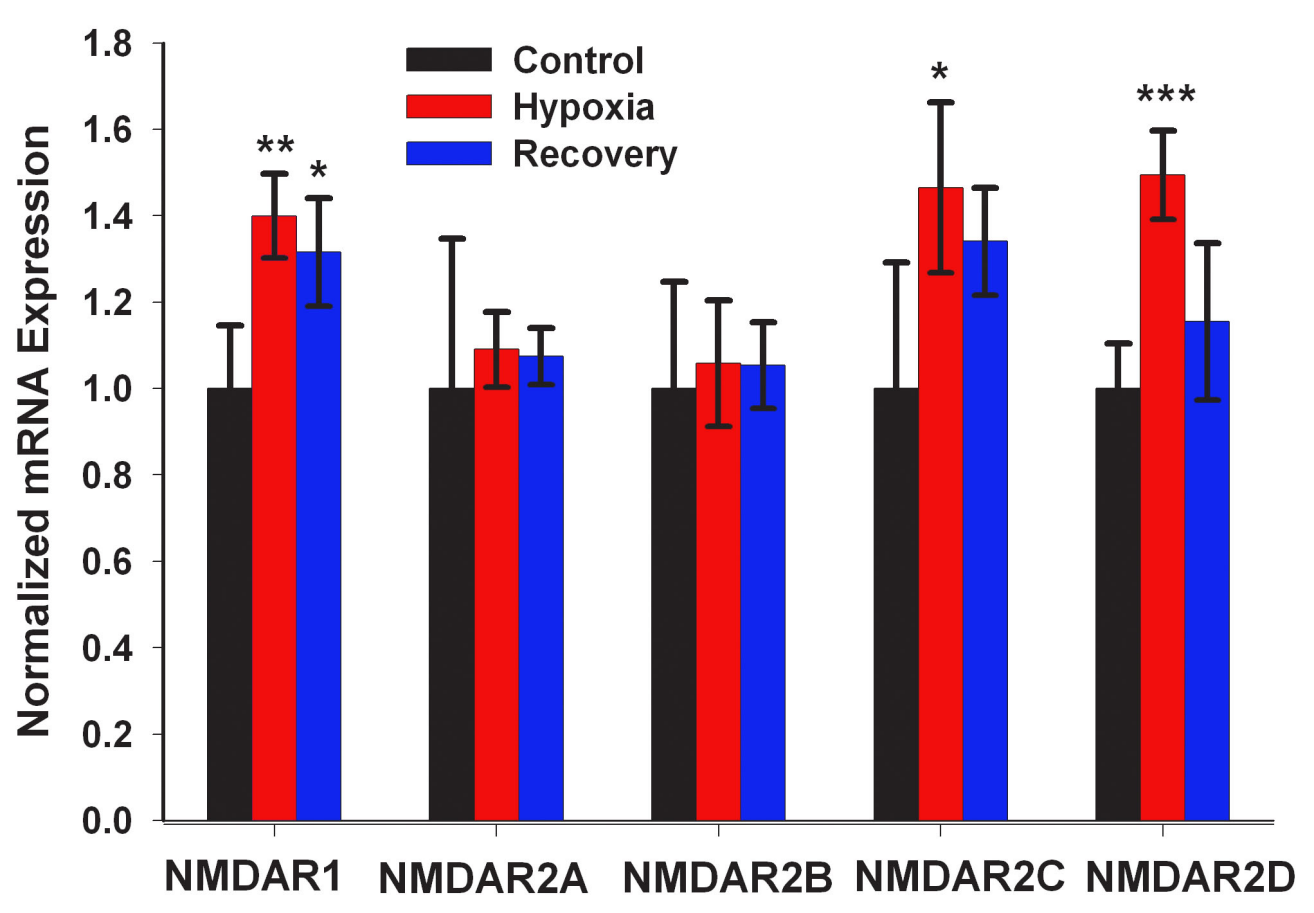
NMDA receptor subunit mRNA expression in the rat retina following hypoxia and 24-h recovery in air. For each mRNA, transcript abundance, normalized to acidic ribosomal protein P0, was measured by qRT–PCR. Values were then further normalized to the control level of each transcript. Data represent mean (±SD) of 5 independent experiments. NMDAR1 mRNA levels were higher in hypoxic samples compared to control and in recovery samples compared to control. NMDAR2C mRNA levels were higher in hypoxia samples compared to the control samples. NMDAR2D mRNA levels were higher in hypoxia samples compared to the control samples. The asterisks indicate significance levels assessed via ANOVA followed by post-hoc tests: * - p<0.05; ** - p<0.01; *** - p<0.001.

The expression pattern of the individual *NMDA-R* subunits (*NR1, NR2A, NR2B, NR2C*, and *NR2D*) was further studied by quantitative real-time RT–PCR. *NR1, NR2C*, and *NR2D* were significantly elevated in hypoxic retinas compared with controls; these results were in close agreement with the microarray results (Figure 4).

### NR1 interactome analysis

**Figure 5:**
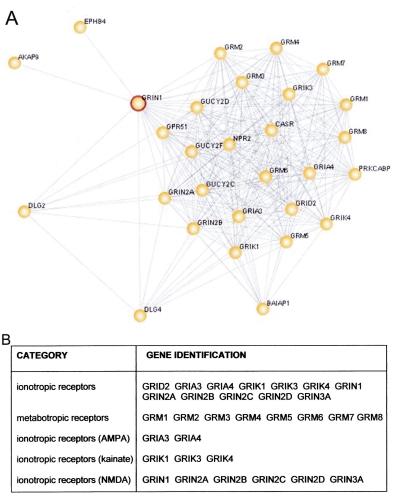
NMDAR1 Interactome. **A:** The molecular network of direct physical, transcriptional, and enzymatic interactions with NMDAR1 (GRIN1), referred to as the NMDAR1 “interactome,” was derived from the HiMAP database. Using evidence from literature-confirmed interactions within the Human Protein Reference Database and predicted interactions generated by Bayesian Analysis, greater than >40,000 molecular relationships were queried. **B:** Subsets of the NMDA Interactome were created based on ligand specificity. In these subsets, the 25 genes in the NMDA interactome (above) were organized into progressively smaller subsets and further analyzed by GSEA.

Insight into the novel role that NMDA receptors play in retinal responses to hypoxia may be derived from study of the coordinated expression patterns of genes that interact, either directly or indirectly, with the NMDA receptor; namely, the NMDA interactome. Initially, the subset of members interacting with NR1, the obligatory subunit of all functional NMDA receptor complexes [57], was identified using the Human Interactome Map (HiMAP) database. HiMAP is a searchable, online browser that allows exploration of both known and predicted protein–protein interactions. Literature-confirmed interactions come from the Human Protein Reference Database, the yeast-two-hybrid-defined interactions come from two recent publications [58,59] and predicted interactions were generated by a Bayesian Analysis [60] From among the >40,000 interactions in the HiMAP network, 32 genes that interact with the NMDAR1 subunit (specifically, the NMDAR1 interactome) were identified (Figure 5A). We found 22 of these genes were represented on our focused array (*GPR51, GRIA3, GRIA4, GRID2, GRIK 1, GRIK3, GRIK4, GRIN1, GRIN2A GRIN2B, GRIN2C, GRIN2D, GRIN3A, GRM1, GRM2, GRM3, GRM4, GRM5, GRM6, GRM7, GRM8*, and *GUCY2F*). Ten were not represented (*AKAP9, BAIAP1, CASR, DLG2, EPHB4, GRIN3B, GUCY2C, GUCY2D, NPR2*, and *PRKCABP*). We used this 22 gene-defined *NMDAR1* interactome to calculate the statistical enrichment of this geneset using GSEA. As a whole, there was no statistically significant enrichment of this geneset. If, however, we partitioned this geneset into discrete subsets (of ≥2 genes) based on ligand specificity (see Categories in Figure 5B), along with the expected significant enrichment of NMDA receptors (p<0.05), there was also significant enrichment in the expression of the metabotropic receptors following hypoxia and recovery (p<0.01).

## Discussion

The purpose of this study was to identify changes in gene expression induced by hypoxia in rat retina both immediately following hypoxia as well as following a 24 h recovery period post hypoxia. To do so, we used an in-house-fabricated, focused microarray platform, detailed in Kroes et al. [47], that deserves further elaboration. The quality of our platform has been rigorously evaluated in terms of dynamic range, discrimination power, accuracy, reproducibility, and specificity. The ability to reliably measure even low levels of statistically significant differential gene expression stems from coupling a) stringently designed and quality controlled chip manufacturing and transcript labeling protocols; b) rigorous data analysis algorithms; and c) flexible ontological and interactome analyses (the bioinformatics tools GoMiner, GSEA, and HiMAP-based interactome analysis) capable of demonstrating significant correlations between the expression of specific genesets. Used together, these technologies provide maximal statistical rigor to analyses of coregulation of genesets that are functionally related or related by involvement in a given biologic pathway. When combined with robust qRT–PCR corroboration, this approach provides a powerful platform to identify fundamental, biologically relevant gene families significantly altered in the hypoxic retina.

Many studies have demonstrated that meaningful expression patterns can involve groups of transcripts whose relative abundance changes at levels considerably less than twofold [61,62]. Additionally, the interpretation of levels of change in gene expression in isolation, without the additional statistical rigor provided by the concomitant analysis of ontological interaction networks, may severely limit critical insights into relevant biologic processes. Thus, interactome hubs such as NR1 may exhibit low levels of change in individual gene expression following hypoxia, but, based on analysis of interaction networks, are likely to play an important role in regulating the biologic response.

From a more global perspective, 22 gene families, of the over 200 identified, were found to be significantly altered by hypoxia. In particular, alterations in genes associated with response to stress, apoptosis, ion channel activity, and neuronal and synaptic modeling were observed. After 24 h of recovery from hypoxia, similar gene families were altered as well. Most (72%) of the genes overexpressed due to hypoxia remained overexpressed after 24 h of recovery, and 50% of the genes that showed a reduction in expression due to hypoxia remained at significantly lower levels of expression than non-hypoxic controls after 24 h of recovery. It therefore is clear that after 24 h of recovery, the retina remains markedly different than pre-hypoxic controls in terms of gene expression patterns. It is also noteworthy that neither overexpressed genes nor genes reduced in expression compared to controls ever showed the opposite patterns of expression in the 24 h recovery retinas. Apart from the *EPO-EPOR* changes, which are probably protective, it is not clear in most cases which gene changes help the retina survive hypoxia, and which may participate in detrimental effects such as neovascularization and apoptosis. However, within the group that remain altered after 24 h are some genes that may underly the positive effects of hypoxic preconditioning [43,63].

Our results confirmed and extended previous observations that genes associated with hypoxia (specifically *VEGF, Flk-1*, and *EPO*) are indeed elevated following hypoxia in the retina. However, hypoxia-associated genes, *Flt-1* and *EPOR*, showed no significant changes in the current study. Interestingly, after 24 h of recovery from hypoxia *VEGF, Flk-1*, and *EPO* returned to control levels of expression, but *EPOR* was significantly elevated. Consistent with this finding, EPOR protein levels continued to rise for 72 h after the end of ischemia in rat retina [64]. *EPO* has now been shown to be protective against ischemic injury [64] and light damage [16], and the upregulation of *EPOR* following a recovery period post-hypoxia is likely important in this protection.

We found a substantial increase in HIF-1 protein during hypoxia, but this does not mean that HIF-1 alone regulated gene expression. Most of the genes that were upregulated during hypoxia in our study are not known to be controlled by HIF-1. Wenger et al. [65] compiled a list of the 70 genes that were known to be regulated directly by HIF-1. This may not be an exhaustive list of the HIF-1 regulated genes, but only a few of the genes that were altered in our study are on that list. Even for those that are under the control of HIF-1, upregulation may require additional factors as well [65]. It should also be noted that hypoxia can act on transcription without involving HIF-1. Among the other possible pathways that have been described as mediating hypoxic responses are those using jun, AP-1, calmodulin, and MAP kinase as intermediates [66].

There have been several recent reports in which some of the same transcripts have been altered by hypoxia or manipulations involving hypoxia. Yoshimura et al. [67] used microarrays to examine gene expression in rat retinal ischemia-reperfusion injury. Retinal expression profiles were investigated 12 h after a 1 h ischemic episode and compared to non-ischemic controls. At this time point, 135 genes and expressed sequence tags (ESTs) were significantly upregulated or downregulated relative to control. Upregulated genes clustered into at least 7 functional groups, including immediate early genes or transcription factors, cell-cycle related genes, stress-responsive protein genes, cell signaling protein genes, cell adhesion and cell surface protein genes, genes involved in translation and protein turnover, and genes encoding metabolic proteins. Kaur et al. [68] examined selected gene expression patterns in rat retinas from 3 to 14 days after hypoxia. mRNAs and protein expression for *HIF-1α, VEGF*, *nitric oxide synthase*, and glutamate receptor subunits *NMDAR1, GluR2, GluR3* were each found to be upregulated at 3 h and 24 h, but all returned to control levels by 14 days.

Several studies have investigated the genomic response of the brain to hypoxic preconditioning. Tang et al. [63] identified differential expression in hypoxia. Several genes were identified as upregulated after 1 h of hypoxia including adenosine receptor *A2AR*, and another group of genes was increased after 6 h of hypoxia including *VEGF, adrenomedullin,* and *GLUT-1*. Similarly, Bernaudin et al. [43] found increased expression of 18 genes in the neonatal rat brain following hypoxia (8% O_2_ for 3 h) including several known hypoxia inducible genes such as *MAP kinase phosphatase-1 (MKP-1)*, several HIF-1 target genes including *VEGF* and *GLUT-1*, genes implicated in apoptosis, signal transduction molecules, and transcription factors. Several novel hypoxia inducible genes, such as a calcium-activated potassium channel (AF083341) and a voltage-dependent potassium channel (X12589), were upregulated. Interestingly, it has been suggested that the initial signal responsible for triggering the development of hypoxic preconditioning in the brain involves the opening of ATP-sensitive potassium channels via the activation of adenosine A1 receptors [69]. In the present study, we identified the modulation of several K^+^ ion channels in addition to *adenosine A3 receptors* during both hypoxia and recovery.

Recently, in investigating preconditioning in the retina, Thiersch et al. [32] reported microarray results for mouse at different time points after 6 h of severe hypoxia (6% O_2_). Using an Affymetrix microarray platform, they found 431 genes significantly altered immediately after hypoxia (of approximately 39,000 transcripts on the Mouse Genome 430 2.0 array); in contrast to the present results in which the effect of hypoxia was persistent, only 3 transcripts were significantly altered after 16 h of recovery.

We chose to investigate the expression of *NMDARs* in more detail. This receptor-ionophore complex is well established as playing a significant role in excitotoxic damage to retinal neurons [38,40,70]. The precise role of NMDARs in ganglion cell death remains somewhat controversial, because one recent study, using cultured neonatal retinal ganglion cells, showed that ganglion cells, in contrast to amacrine cells, were not killed by NMDA [71]. Nevertheless, retinal hypoxia induced by glutamate-mediated excitotoxicity is thought to be a key antecedent to such retinal diseases as glaucoma and diabetic retinopathy [39,72,73].

The NMDA receptor subtype mRNAs *for NR1, NR2A-D*, and *NR3* are expressed in mouse retinal ganglion and amacrine cells [74]. Only *NR1* mRNA expression could be detected in bipolar cells, and no NMDAR subunit mRNA expression was detected in rods [74]. To provide additional information on NMDARs, we used the following: 1) GSEA; 2) qRT–PCR; and 3) interactome analysis using HiMAP. First, GSEA analysis showed a nearly sixfold enrichment in NMDAR both after hypoxia and 24 h after recovery. Kaur et al. [68] found a maximal elevation of about twofold in the *NR1* subunit of the NMDAR complex after 24 h of hypoxia using qRT–PCR. Second, in our model, qRT–PCR analysis of *NR1* expression showed about a 30% increase over controls that was also significant (p<0.01). We also found significant increases in *NR2C* and *NR2D* subunits, but not in *NR2A* or *NR2B*. Interestingly, only the *NR1* subunit remained significantly elevated after 24 h of recovery from hypoxia, so these transcripts exhibit differential responsiveness to hypoxia than other NR subunits. Third, HiMAP interactome analysis coupled with GSEA analysis allowed us to extend our study to all of the genes that NR1 receptors directly interact with. These analyses revealed that expression of the metabotropic glutamate receptor family was also significantly enriched following hypoxia and recovery

Our results focused on mRNA expression and not protein expression or functional NMDAR changes after hypoxia and recovery. Nevertheless these results may have important implications for the design of retinal neuroprotective agents because they suggest that NR2C and NR2D-containing NMDAR subtypes may be the best targets. It has been noted that NMDAR antagonists would not be good candidates for neuroprotection because of the need to maintain retinal function for normal visual information processing. Interestingly, it has recently been shown that memantine, an uncompetitive NMDA receptor antagonist that inhibits overactivity of NMDARs, was able to prevent retinal ganglion cell degeneration in streptozotocin-induced diabetic rats [73]. Perhaps partial agonists, directed at these receptor subtypes will have the greatest neuroprotective potential for they would have the ability to inhibit excessive receptor activity without totally shutting down the receptor’s ability to perform normal neuronal functions. Two such partial agonists with clinical potential are D-cycloserine and GLYX-13 [75,76].

## Appendix 1. Hypoxia-associated gene expression in the rat retina.

To access the data, click or select the words “Appendix 1.” This will initiate the download of a PDF file. Categories are convenient functional groupings and are not related to GO categories. The fold change was calculated between mean values of hypoxic retinas (n=6) and control normoxic (n=6) retinas in the hypoxia column and between mean values from retinas after 24 h of recovery from hypoxia (n=6) and control normoxic retinas (n=6) in the recovery column. Positive values indicate an increase and negative values indicate a decrease in gene expression relative to control retina. No significant change is indicated by nc. RGD represents Rat Genome Database.
